# A Recombinant Subunit Vaccine Against Chicken Infectious Anemia Virus Elicits Protective Immunity via VP2-Assisted VP1 Refolding

**DOI:** 10.3390/vaccines14040292

**Published:** 2026-03-25

**Authors:** Shihao Li, Mingxue Hu, Yanping Zhang, Yulu Duan, Ru Guo, Huijing Sun, Wenzhuo Ma, Xiaole Qi, Hongyu Cui, Suyan Wang, Yuntong Chen, Yongzhen Liu, Yulong Gao

**Affiliations:** Avian Immunosuppressive Diseases Division, State Key Laboratory of Animal Disease Control and Prevention, Harbin Veterinary Research Institute, Chinese Academy of Agricultural Sciences, Harbin 150001, China

**Keywords:** chicken infectious anemia virus, VP1, VP2, immunity, subunit vaccine

## Abstract

Background: Chicken infectious anemia virus (CIAV) is a globally significant immunosuppressive pathogen that causes substantial economic losses to the poultry industry, with particularly severe outbreaks in China in recent years. Given the limitations of existing vaccines, especially the residual virulence associated with live attenuated vaccines, there is an urgent need to develop novel, safer, and more effective vaccine strategies. Methods: In this study, the VP1 and VP2 genes of CIAV were cloned and expressed in *Escherichia coli* to develop a cost-effective subunit vaccine. Since VP1 primarily formed inclusion bodies, a “VP2-assisted co-refolding” strategy was employed. This involved denaturing VP1 and refolding it via gradient dialysis in the presence of soluble VP2, thereby leveraging VP2’s natural chaperone-like function to restore conformational epitopes. The refolded VP1/VP2 protein complexes, emulsified at different ratios, were used to immunize 3-day-old specific pathogen-free (SPF) chickens, followed by challenge with a virulent CIAV strain. Results: The vaccine formulation with a VP1:VP2 ratio of 1:1 provided the best protection, achieving 71.4% (5/7) protective efficacy, as evidenced by significantly reduced thymic atrophy and a higher thymus index. Conclusions: These findings validate the feasibility of using an economical prokaryotic expression system combined with a rational protein refolding strategy to produce a protective subunit vaccine candidate against CIAV, offering a promising alternative for disease control.

## 1. Introduction

Chicken infectious anaemia (CIA) is an immunosuppressive disease caused by the chicken infectious anaemia virus (CIAV) [1]. Since its initial identification in Japan in 1970, CIAV has become distributed worldwide [2]. In recent years, China has faced particularly severe outbreaks driven by the emergence of highly pathogenic strains, making the virus a major threat to its poultry industry [3]. The virus infects the bone marrow hematopoietic cells and pre-T lymphocytes in the thymus of chicks [4], leading to anaemia and immunosuppression in chickens typically exhibiting yellowish-white bone marrow, atrophy of thymic lymphoid tissue, and hemorrhagic lesions [5]. Beyond these clinical signs, CIAV infection induces extensive apoptosis of thymocytes [6], which profoundly impairs the generation of pathogen-specific cytotoxic T lymphocytes [7]. In atrophied thymuses, hematopoietic cells are gradually replaced by adipocytes or proliferating stromal cells [8], while the cortex and medulla are progressively replaced by proliferating reticular cells and fibroblasts. CIAV infection often occurs concurrently with, exacerbates, or complicates infections caused by other viruses, bacteria, or fungi, posing a serious threat to the health of chicken flocks [9]. CIAV has been shown to suppress vaccinal immunity against other diseases, such as Marek’s disease [10], further increasing economic risks.

Currently, commercially available live attenuated vaccines are widely used in breeder flocks outside of China [11]. However, these vaccines retain residual virulence and can cause damage to the immune organs of chickens [8]. Specifically, vaccinal strains have been reported to persist in the spleen and thymus of young chicks, inducing thymic lymphoid cell disorders and causing significant atrophy of lymphoid tissues, which may compromise the host’s immune status [8]. Although inactivated vaccines offer moderate protection for breeders [12], their large-scale commercial production is hindered by the inability to propagate CIAV to high titers in vitro [13]. While innovative strategies such as DNA vaccines [14] or virus-like particles (VLPs) [15] have been investigated to address these issues, they often face challenges related to high production costs and complex manufacturing processes. Consequently, the development of safer and more efficacious vaccines is crucial for the effective control of CIAV.

The viral genome of CIAV contains three overlapping open reading frames (ORFs) [16]. These ORFs encode three functionally distinct proteins: VP1, VP2, and VP3. VP1 is the major capsid protein. It possesses DNA-binding activity [17] and contains a hypervariable region located at amino acids 139–151 [18]. However, VP1 expressed in isolation often fails to adopt its native immunogenic conformation, especially in prokaryotic systems [18]. VP2 acts as a scaffold protein that assists in the proper folding of VP1 [19]. It also exhibits dual-specificity phosphatase activity [20], which is essential for viral replication. VP3 localizes to the nucleus of infected cells and functions to induce apoptosis [21]. Based on these functional attributes, the co-expression or combined use of VP1 and VP2 has emerged as a rational strategy for developing genetically engineered vaccines against CIAV [22].

In this study, we aimed to address a key challenge in developing a cost-effective CIAV subunit vaccine by leveraging a prokaryotic expression system. The central problem was that the major capsid protein VP1, when expressed in *E. coli*, predominantly misfolds into insoluble inclusion bodies, likely compromising its native immunogenic structure. Based on the known chaperone-like function of VP2, we hypothesized that co-refolding denatured VP1 in the presence of soluble VP2 in vitro could restore VP1’s protective conformational epitopes. This “VP2-assisted co-refolding” strategy leverages VP2’s natural function to restore critical conformational epitopes. Furthermore, we sought to empirically determine the optimal stoichiometric ratio of VP1 to VP2 for this process by evaluating complexes formed at different molar ratios. The immunogenicity and protective efficacy of the refolded protein complex were subsequently evaluated in a chicken model, providing a promising and economical alternative for the prevention and control of CIAV.

## 2. Materials and Methods

### 2.1. Animals and Ethical Statement

Specific pathogen-free (SPF) chickens used in this experiment were obtained from the National Poultry Animal Laboratory Resource Centre (Harbin, China). This study was conducted strictly in accordance with the recommendations of the Guide for the Care and Use of Laboratory Animals. Experimental chickens were anesthetized via CO_2_ inhalation prior to dissection, then euthanised by exsanguination. Thymus tissues were collected and examined during necropsy. All animal experiments were approved by the Animal Ethics Committee of the Harbin Veterinary Research Institute (HVRI) and conducted under an authorised scheme (No. 241206-03-GR, 6 December 2024).

### 2.2. Viruses and Plasmids

The virus isolated from liver samples of diseased chickens at a Jilin Province farm was cultured in RPMI-1640 medium supplemented with 10% fetal bovine serum. MDCC-MSB1 cells (maintained in our laboratory) were cultured at 37 °C under 5% CO_2_ conditions. Following inoculation, the virus underwent serially passaged until pronounced viral replication characteristics emerged. This isolate was ultimately designated CIAV JL17P10 strain and used as the challenge virus for subsequent experiments. The HeN/193001 strain, obtained from a poultry farm in Henan Province using the same method, served as the parental virus and template for subsequent PCR amplification sequences. The complete genomes of both isolated viruses were ligated to the pMD™ 18-T vector (Takara, Beijing, China) for sequencing validation, yielding the full viral sequences.

### 2.3. Construction of the Expression Plasmid and VP1, VP2 Proteins

The nucleic acid sequences of CAIV VP1 and VP2 were optimised for *E. coli* codons and synthesised by Harbin Seven Bioscience Co., Ltd. (Harbin, China) in the pGEX-6P-1 prokaryotic expression vector. The prokaryotic expression vectors pET32a and pET24b were obtained from laboratory stocks. The full-length VP1 gene sequence was amplified using primers VP1-F1 and VP1-R1, and inserted into pET32a vector digested with restriction enzymes BamHI (NEB, Beijing, China) and XhoI. Homologous recombination ligation with C112 (Takara, Beijing, China) for homologous recombination to construct the complete plasmid. The vector carries an N-terminal 6 × His tag to facilitate SaI1 purification. The entire VP2 gene sequence was amplified using primers VP2-F1 and VP2-R2 and inserted into the pET24b vector digested with restriction enzymes SalI and XhoI. A Sumo tag was introduced at the C-terminus of the construct to promote soluble expression. Upon completion, the two plasmids were named pET32a-VP1 and pET24b-VP2, respectively. The primers employed in the aforementioned experiments are presented in Table 1. Following plasmid construction, pET32a-VP1 was verified via double digestion with restriction endonucleases BamHI and XhoI under the following conditions: 5 μL 10× Buffer, 1 μL BamHI, 1 μL XhoI, 5 μg plasmid DNA, supplemented with double-distilled water to a total volume of 50 μL. Verification of pET24b-VP2 was performed using a double digestion with SalI and XhoI using the same reaction system. PCR amplification of pET32a-VP1 and pET24b-VP2 was performed using primer pairs VP1-F1/VP1-R1 and VP2-F1/VP2-R1, respectively. Successful construction of VP1 and VP2 into the prokaryotic vectors was confirmed by agarose gel electrophoresis.

### 2.4. Expression and Purification of VP1 and VP2 Proteins

The constructed plasmids pET32a-VP1 and pET24b-VP2 were transformed into *Escherichia coli* BL21 (DE3) cells (WEIDI, Shanghai, China) to induce expression of the VP1 and VP2 proteins. The *E. coli* strains harbouring these two plasmids were inoculated into lysogeny broth (LB) containing the corresponding antibiotic. Cultures were incubated at 37 °C with shaking at 220 rpm for 2–3 h until an OD_600_ of 0.8 was reached, at which point 1 mM IPTG was added for induction. Cultures were then transferred to shaking incubators maintained at 16 °C, 20 °C, and 27 °C, respectively. Following incubation, bacteria were harvested from lysogeny broth medium and resuspended in PBS. The suspension was subjected to pulsed ultrasonication at 39% amplitude on ice until clarification. The lysate was centrifuged at 12,000× *g* for 30 min to separate the supernatant and inclusion body pellet. Specified volumes of each fraction were mixed with 5× loading buffer and boiled for 10 min. Protein expression was confirmed by SDS-PAGE and Western blot analysis.

After determining the optimal expression temperature, large-scale protein expression was performed. A 1:100 dilution of the bacterial culture was inoculated into 500 mL of LB for induction. Following centrifugation to collect the cells, the pellet was resuspended in Binding Buffer (20 mM sodium phosphate, 500 mM sodium chloride, 30 mM imidazole, pH 7.4). After ultrasonic disruption as described above, the supernatant and inclusion bodies were separated. The VP1 inclusion body pellet was dissolved in 8 M urea to form a clear solution for subsequent use. For the VP2 protein, the soluble fraction was purified by Ni-NTA affinity chromatography. The supernatant obtained after ultrasonication was loaded onto a Ni-NTA resin column and incubated overnight at 4 °C to ensure maximum binding. Gravity flow purification was performed. The Ni-NTA resin bound with VP1 protein (from solubilized inclusion bodies) was washed three times with 5 column volumes (CV) of Binding Buffer containing 50 mM imidazole. Elution was performed with 1 CV of Elution Buffer (20 mM sodium phosphate, 500 mM NaCl, 500 mM imidazole, pH 7.4) to obtain purified VP1 protein. The Ni-NTA resin bound with VP2 protein was washed three times with 5 CV of Binding Buffer containing 90 mM imidazole and eluted with 1 CV of Elution Buffer to obtain purified VP2 protein. GST-tagged VP1 protein was expressed using the pGEX-6P-1 vector. The supernatant was loaded onto Glutathione Sepharose 4B resin and incubated overnight at 4 °C. After washing, 1 CV of Glutathione Elution Buffer (10 mM reduced glutathione, 50 mM Tris-Cl, pH 8.0) was added to the resin, and the mixture was gently agitated at room temperature for 10 min to elute the bound protein. The eluate was collected by centrifugation at 500× *g* for 5 min at 4 °C. The elution step was repeated three times, and the three eluates were pooled. The concentrations of the three purified proteins were determined using the Bradford method. Purified proteins were stored at −80 °C until further use.

### 2.5. Inclusion Body Protein Gradient Dialysis Reproteinisation

In vitro renaturation of VP1 protein from inclusion bodies was performed using gradient dialysis. Briefly, the partially purified inclusion body protein was fully solubilized in denaturing buffer containing 8 M urea. The entire protein solution was then transferred into a pretreated dialysis bag (Biosharp, Guangzhou, China) and sealed tightly. The dialysis bag was sequentially placed in renaturation buffers containing decreasing concentrations of urea for stepwise dialysis: first in 6 M urea solution at 4 °C for 6–8 h to initiate unfolding, followed by transfer to 4 M urea solution for 4 h, during which pre-purified VP2 protein was added to promote heterocomplex assembly. Finally, the bag was transferred to 2 M urea solution and dialyzed overnight (approximately 12–16 h) at 4 °C under gentle stirring to progressively remove the denaturant, allowing the protein to slowly refold into its native conformation. Two experimental groups were established based on the ratio of VP1 to VP2 protein added: a 1:1 group and a 2:1 group. Upon completion of dialysis, samples were collected from the dialysis bags, and protein expression was analyzed by SDS-PAGE.

### 2.6. Vaccine Immunisation and Animal Experiments

Sixty-one SPF White Leghorn (National Poultry Experimental Animal Resource Centre, Harbin, China) were divided into six groups: the control groups comprised ten chickens each in the Negative Control group and Positive Control group; the experimental groups comprised ten chickens each in the VP1 group, 1:1 group, and 3:1 group; and the 2:1 group comprised eleven chickens. Chick mortality prior to immunisation and within 24 h post-immunisation occurred in 2 chickens from the VP1 group, 3 from the 1:1 group, and 4 from the 3:1 group. These were deemed accidental deaths and excluded from subsequent statistical analyses. Among the immunised groups, one group received the purified protein GST-VP1 from Section 2.4, while the other three groups received three different ratios of VP1:VP2 obtained through dialysis reactivation in Section 2.5: 1:1, 2:1, and 3:1. The two mixed protein groups obtained after quantitative dialysis and the GST-VP1 protein were diluted to 100 μg/mL. Each was mixed 1:1 with liquid paraffin oil adjuvant, emulsified by high-speed shearing using a disperser (IKA, Germany), and stored at 4 °C for later use. At three days of age, chickens in the four immunisation groups received a 100 μL intramuscular injection of the oil-adjuvant vaccine into the thigh. Two weeks post-vaccination, four immunised groups were challenged with 10^−5.5^ TCID_50_ of the virulent CIAV strain JL17P10. A fifth group (10 chickens) served as the CIAV challenge control, receiving a subcutaneous injection of 10^−5.5^ TCID_50_ of the virulent CIAV strain JL17P10 on day 21. The sixth group comprised 10 healthy controls receiving the corresponding solvent during the experiment as a sham-inoculation control. Fourteen days post-challenge, thymuses were collected from each group, weighed, and the thymus index was calculated using the following formula: thymus-to-body weight ratio relative to that of the healthy group. A thymus with a TBIX score below 0.80 was considered atrophied.

### 2.7. Statistical Analysis

Data are presented as the mean ± standard deviation (SD). One-way analysis of variance (ANOVA) was conducted to examine differences among multiple groups, followed by independent sample *t*-tests for pairwise comparisons, provided that assumptions of homogeneity of variance and normality were met. All statistical analyses were performed using SPSS (version 17.0; SPSS Inc., Chicago, IL, USA), and a *p*-value < 0.0001 was considered statistically significant.

## 3. Results

### 3.1. Construction of Prokaryotic Expression Vectors and Protein Expression

**Figure 1:**
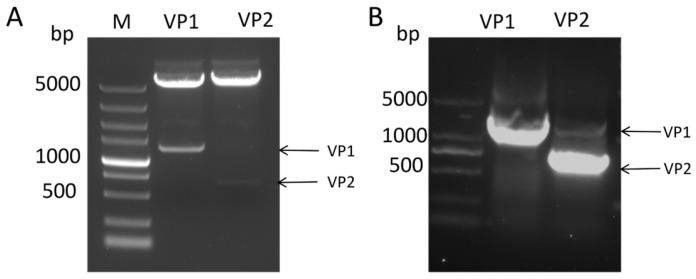
Construction of VP1 and VP2 prokaryotic expression vectors. Identification by restriction enzyme digestion (**A**) and PCR (**B**) confirmed the correct construction of the vectors.

To obtain CIAV VP1 and VP2 proteins, the VP1 and VP2 genes were cloned into the prokaryotic expression vectors pET32a and pET24b, respectively, successfully constructing the recombinant plasmids pET32a-VP1 and pET24b-VP2 (Figure 1A). Identification by restriction enzyme digestion and PCR confirmed the correct construction of the vectors (Figure 1B).

**Figure 2:**
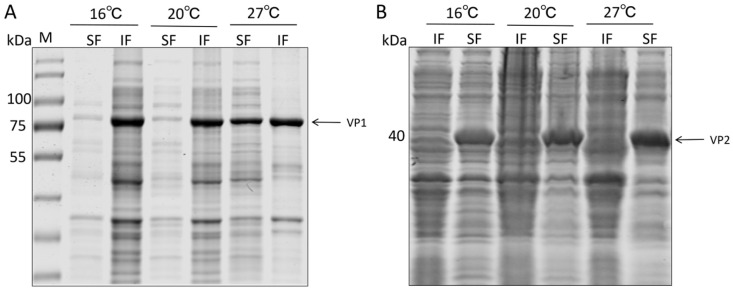
Expression of VP1 and VP2 proteins at different temperatures in the supernatant and inclusion bodies of ultrasonically disrupted *Escherichia coli* cells. SF: Supernatant of ultrasonically disrupted bacterial cells. IF: Inclusion body precipitate after ultrasonic disruption of bacterial cells. (**A**) Expression of pET32a-VP1 at 16 °C, 20 °C, and 27 °C. (**B**) Expression of pET24b-VP2 at 16 °C, 20 °C, and 27 °C.

After transforming these recombinant plasmids into *E. coli* BL21(DE3) competent cells, expression was induced with IPTG at different temperatures (16 °C, 20 °C, and 27 °C). SDS-PAGE analysis revealed distinct specific bands at approximately 72 kDa (VP1) and 53 kDa (VP2, containing the Sumo tag) (Figure 2), which were consistent with the theoretical molecular weights calculated from their amino acid sequences. Expression analysis indicated that the VP1 protein was predominantly expressed as insoluble inclusion bodies at all tested temperatures, with the highest total expression level observed at 27 °C, thus establishing 27 °C as the optimal induction condition for maximizing antigen yield. In contrast, the VP2 protein exhibited favorable soluble expression at 16 °C, 20 °C, and 27 °C, with the highest soluble yield achieved at 20 °C. This high solubility of VP2 was crucial for the subsequent refolding strategy, as it allowed VP2 to function effectively as a chaperone in the liquid phase.

### 3.2. Protein Purification and Refolding

**Figure 3:**
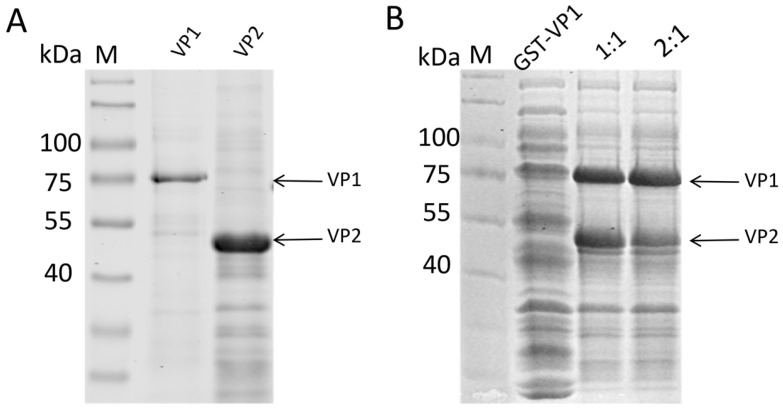
VP1 and VP2 proteins purified using NI affinity chromatography columns. (**A**) VP1 and VP2 proteins purified from the supernatant of ultrasonicated bacterial cells. (**B**) VP1 proteins dissolved in 8 M urea after ultrasonication and VP2 proteins purified and concentrated.

To purify the expressed proteins, nickel-affinity chromatography was first applied to the soluble VP2 fraction, yielding high-purity VP2 protein (Figure 3A). For the VP1 protein, which was primarily present in inclusion bodies, it was first denatured with urea, purified via nickel-affinity chromatography, and then mixed with the soluble VP2 protein at different ratios. Refolding of the mixed proteins was performed using a gradient urea dialysis method to gradually remove the denaturant and allow hydrophobic interactions to re-establish in the presence of the scaffold protein. SDS-PAGE analysis of the refolded protein samples confirmed the successful refolding of both VP1 and VP2 proteins, which remained stable across the different mixing ratios (Figure 3B). Crucially, no significant precipitation was observed in the dialysis bag after the removal of urea, particularly in the mixed groups. This indicates that the interaction between VP1 and VP2 prevented the aggregation of VP1, effectively maintaining it in a soluble state and suggesting the formation of stable VP1/VP2 heterocomplexes.

### 3.3. Protective Efficacy of the VP1/VP2 Subunit Vaccine Against CAV Challenge

**Figure 4:**
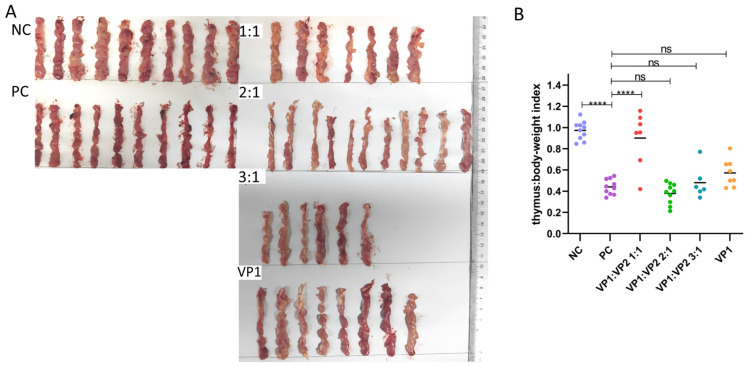
Evaluation of the immunogenicity of subunit vaccines in chickens. Each chicken was immunised with 50 ng, with a blank control group and a challenge control group. (**A**) Thymus tissue was collected from each group after necropsy to observe the degree of thymus atrophy. (**B**) Thymus index two weeks after CAV challenge. ****, *p* < 0.0001; ns, no significant difference.

To evaluate the immunoprotective effect of the refolded VP1/VP2 complex proteins, the proteins were emulsified at different ratios and used to immunize 3-day-old SPF chickens. Following immunization, some chicks in the VP1, 1:1, and 3:1 groups exhibited lameness or died within 24 h, likely due to the technical challenge of intramuscular injection in 3-day-old chickens. These individuals were deemed accidental deaths and excluded from the final analysis. Consequently, protective efficacy was assessed in the following final group sizes: VP1 (*n* = 8), 1:1 (*n* = 7), 2:1 (*n* = 11), and 3:1 (*n* = 6). At 14 days post-immunization, the chickens were challenged with a virulent CAV strain. Necropsy results at 12 days post-challenge showed significant thymic atrophy in both the blank control and challenge control groups, characterized by a marked reduction in thymic lobe size and a change in color from healthy white to translucent or yellowish, indicating severe depletion of lymphoid cells. Among the experimental groups, the VP1:VP2 (1:1) immunized group performed best, with 5 out of 7 chickens showing no macroscopic thymic lesions and retaining normal thymic morphology. Statistical analysis revealed that the protection rate of the 1:1 group (71.4%) was significantly higher than that of the challenge control group. In contrast, the other VP1:VP2 ratio groups and the VP1 protein control group exhibited varying degrees of thymic atrophy, demonstrating limited protective efficacy (Figure 4A). This disparity highlights the critical role of the stoichiometric ratio in forming effective immunogens. Thymus index analysis further indicated that only the VP1:VP2 (1:1) immunized group had a thymus index significantly higher than that of the challenge control group (Figure 4B), suggesting that this formulation effectively protected the immune organs from virus-induced damage. These results demonstrate that, among all tested formulations, the subunit vaccine prepared at a 1:1 ratio of VP1 to VP2 proteins provided the optimal protective efficacy, achieving a protection rate of 71.4% (5/7) against challenge with the virulent CAV strain. This protection rate was superior to that of the VP1-only group, confirming that the VP2-assisted refolding strategy significantly enhances the immunogenicity of the subunit vaccine.

## 4. Discussion

The development of a vaccine that balances high safety with economic feasibility remains the central challenge in controlling CIAV [1,2,3]. While current live and inactivated vaccines have reduced disease incidence, they are persistently limited by safety risks regarding virulence reversion or prohibitive production costs [8,11,12,13]. In this study, we established a scalable prokaryotic platform that successfully overcomes the inclusion body bottleneck via a novel “VP2-assisted co-refolding” strategy. Our data demonstrate that the refolded VP1/VP2 complex not only recovers native immunogenicity but also confers significant protection (71.4%) in clinical challenge, providing a solid proof-of-concept for a safer, cost-effective next-generation subunit vaccine.

A key finding of this study was the critical dependence of vaccine efficacy on the stoichiometric ratio of VP1 to VP2. Our gradient design (1:1, 2:1, and 3:1) revealed that the 1:1 ratio elicited superior protection compared to the VP1-excess groups (2:1 and 3:1) and the VP1-only control. We propose that this enhanced efficacy is driven by two immunological mechanisms. First, sufficient VP2 likely acted as a chaperone to facilitate the exposure of conformational neutralizing epitopes on VP1, which are otherwise buried in misfolded inclusion bodies. These epitopes are essential for triggering humoral immunity to block viral entry. Second, the formation of stable, particulate VP1/VP2 heterocomplexes may have facilitated antigen uptake and presentation by dendritic cells, potentially promoting a Th1-biased cellular immune response (e.g., IFN-γ production) necessary for clearing infected cells [23]. The reduced protection in the 2:1 and 3:1 groups confirms that sub-stoichiometric amounts of VP2 fail to prevent hydrophobic aggregation, leading to the loss of these critical structural features.

While this study establishes the feasibility of the VP2-assisted refolding strategy, it represents a preliminary proof-of-concept, and several limitations must be addressed in future work. First, our evaluation relied primarily on clinical and pathological endpoints (protection rate and thymus index). Therefore, a detailed characterization of the immune response is essential. Future studies will systematically quantify virus-neutralizing antibody titers and analyze cellular immune markers (e.g., IL-12, IFN-γ, and lymphocyte proliferation) to fully elucidate the correlates of protection. Second, regarding process optimization, we focused on screening ratios where VP1 was in excess or equal to VP2. Future optimization will verify whether a molar excess of VP2 (e.g., a 1:2 ratio) could further enhance the yield or stability of the refolded complexes.

Looking forward, the pivotal future direction lies in advanced antigen engineering to assemble Virus-Like Particles (VLPs). Unlike soluble complexes, VLPs mimic the native virion’s T = 1 icosahedral symmetry, presenting high-density conformational epitopes that effectively cross-link B-cell receptors to trigger potent neutralizing antibodies and cellular immunity [15]. The feasibility of this approach is strongly bolstered by the success of *E. coli*-derived vaccines for Porcine Circovirus Type 2 (PCV2), a virus structurally analogous to CIAV [24].

## 5. Conclusions

Studies confirm that prokaryotic PCV2 VLPs can induce robust humoral and cellular immune responses and provide protection comparable to commercial vaccines [25,26,27]. Therefore, advancing the research toward a VLP-based vaccine strategy represents a significant forward-looking direction. Combining the cost-efficiency of our *E. coli* expression system with optimized VLP assembly technology could ultimately yield a superior, next-generation vaccine that perfectly balances safety, efficacy, and affordability for global CIAV control.

## Figures and Tables

**Table 1:** Primers used in this study.

Primer Name	Primer Sequence (5′-3′)
VP1-F1	GCCATGGCGATATCGGATCCGATGGCACGTCGTGCACGTCG
VP1-R1	AGTGGTGGTGGTGGTGGTGGTGGTGCTCGAGCGGCTGGCTACCCCA
VP2-F1	GGATCCGAATTCGAGCTCCGTCGACATGCACGGTAACGGC
VP2-R1	CATCGGACACCTTTAAATTGATGTGAGTCTCAGGC

## Data Availability

Data can be requested by writing to the author.

## References

[1] McNulty M.S. (1991). Chicken anaemia agent: A review. Avian Pathol..

[2] Techera C., Marandino A., Tomás G., Grecco S., Hernández M., Hernández D., Panzera Y., Pérez R. (2021). Origin, spreading and genetic variability of chicken anaemia virus. Avian Pathol..

[3] Fang L., Jia H., Hu Y., Wang Y., Cui Z., Qi L., Zhao P. (2023). Molecular characterization and pathogenicity study of a highly pathogenic strain of chicken anemia virus that emerged in China. Front. Cell. Infect. Microbiol..

[4] Noteborn M.H., de Boer G.F., van Roozelaar D.J., Karreman C., Kranenburg O., Vos J.G., Jeurissen S.H., Hoeben R.C., Zantema A., Koch G. (1991). Characterization of cloned chicken anemia virus DNA that contains all elements for the infectious replication cycle. J. Virol..

[5] Wani M.Y., Dhama K., Malik Y.S. (2016). Impact of virus load on immunocytological and histopathological parameters during clinical chicken anemia virus (CAV) infection in poultry. Microb. Pathog..

[6] Jeurissen S.H., Wagenaar F., Pol J.M., van der Eb A.J., Noteborn M.H. (1992). Chicken anemia virus causes apoptosis of thymocytes after in vivo infection and of cell lines after in vitro infection. J. Virol..

[7] Markowski-Grimsrud C.J., Schat K.A. (2003). Infection with chicken anaemia virus impairs the generation of pathogen-specific cytotoxic T lymphocytes. Immunology.

[8] Vaziry A., Silim A., Bleau C., Frenette D., Lamontagne L. (2011). Chicken infectious anaemia vaccinal strain persists in the spleen and thymus of young chicks and induces thymic lymphoid cell disorders. Avian Pathol..

[9] Bhatt P., Shukla S.K., Mahendran M., Dhama K., Chawak M.M., Kataria J.M. (2011). Prevalence of chicken infectious anaemia virus (CIAV) in commercial poultry flocks of northern India: A serological survey. Transbound. Emerg. Dis..

[10] Zhang Y., Cui N., Han N., Wu J., Cui Z., Su S. (2017). Depression of vaccinal immunity to Marek’s disease by infection with chicken infectious anemia virus. Front. Microbiol..

[11] Rosenberger J.K., Cloud S.S. (1989). The isolation and characterization of chicken anemia agent (CAA) from broilers in the United States. Avian Dis..

[12] Zhang X., Wu B., Liu Y., Chen W., Dai Z., Bi Y., Xie Q. (2015). Assessing the efficacy of an inactivated chicken anemia virus vaccine. Vaccine.

[13] Huynh L.T.M., Nguyen G.V., Do L.D., Dao T.D., Le T.V., Vu N.T., Cao P.T.B. (2020). Chicken infectious anaemia virus infections in chickens in northern Vietnam: Epidemiological features and genetic characterization of the causative agent. Avian Pathol..

[14] Sawant P.M., Dhama K., Rawool D.B., Wani M.Y., Tiwari R., Singh S.D., Singh R.K. (2015). Development of a DNA vaccine for chicken infectious anemia and its immunogenicity studies using high mobility group box 1 protein as a novel immunoadjuvant. Vaccine.

[15] Tseng T.-Y., Liu Y.-C., Hsu Y.-C., Chang P.-C., Hsieh M.-K., Shien J.-H., Ou S.-C. (2019). Preparation of chicken anemia virus (CAV) virus-like particles and chicken interleukin-12 for vaccine development using a baculovirus expression system. Pathogens.

[16] Koch G., van Roozelaar D.J., Verschueren C.A., van der Eb A.J., Noteborn M.H. (1995). Immunogenic and protective properties of chicken anaemia virus proteins expressed by baculovirus. Vaccine.

[17] Lai G.-H., Lin M.-K., Lien Y.-Y., Cheng J.-H., Sun F.-C., Lee M.-S., Chen H.-J., Lee M.-S. (2018). Characterization of the DNA binding activity of structural protein VP1 from chicken anaemia virus. BMC Vet. Res..

[18] Lee M.-S., Hseu Y.-C., Lai G.-H., Chang W.-T., Chen H.-J., Huang C.-H., Lee M.-S., Wang M.-Y., Kao J.-Y., You B.-J. (2011). High yield expression in a recombinant E. coli of a codon optimized chicken anemia virus capsid protein VP1 useful for vaccine development. Microb. Cell Fact..

[19] Lai G.-H., Lin M.-K., Lien Y.-Y., Fu J.-H., Chen H.-J., Huang C.-H., Tzen J.T., Lee M.-S. (2013). Expression and characterization of highly antigenic domains of chicken anemia virus viral VP2 and VP3 subunit proteins in a recombinant *E. coli* for sero-diagnostic applications. BMC Vet. Res..

[20] Peters M.A., Jackson D.C., Crabb B.S., Browning G.F. (2002). Chicken anemia virus VP2 is a novel dual specificity protein phosphatase. J. Biol. Chem..

[21] Pallister J., Fahey K.J., Sheppard M. (1994). Cloning and sequencing of the chicken anaemia virus (CAV) ORF-3 gene, and the development of an ELISA for the detection of serum antibody to CAV. Vet. Microbiol..

[22] Shen S.Y., Chang W.C., Yi H.H., Tsai S.-S., Liu H.J., Liao P.-C., Chuang K.P. (2015). Development of a subunit vaccine containing recombinant chicken anemia virus VP1 and pigeon IFN-γ. Vet. Immunol. Immunopathol..

[23] Liu L., Yin M., Li Y., Su H., Fang L., Sun X., Chang S., Zhao P., Wang Y. (2022). DNA prime and recombinant protein boost vaccination confers chickens with enhanced protection against chicken infectious anemia virus. Viruses.

[24] Patterson A.R., Opriessnig T. (2020). Epidemiology and vaccine efficacy of porcine circovirus type 2d (PCV2d). Vaccines.

[25] Zhang S., Li Y., Liu J., Cao J., Li X., Wang Z. (2016). Efficient expression and purification of porcine circovirus type 2 virus-like particles in Escherichia coli. Protein Expr. Purif..

[26] Wang N., Zhang M., Xu J., Wang Z., Li Y. (2024). Optimized production of full-length PCV2d virus-like particles in Escherichia coli: A cost-effective and high-yield approach for potential vaccine antigen development. Vaccine.

[27] Wu P.C., Lin W.L., Wu C.M., Wu C.J., Chi J.N., Chien M.S., Huang C. (2012). Characterization of porcine circovirus type 2 (PCV2) capsid particle assembly and its application to virus-like particle vaccine development. Appl. Microbiol. Biotechnol..

